# Photoionisation detection of a single Er^3+^ ion with sub-100-ns time resolution

**DOI:** 10.1093/nsr/nwad134

**Published:** 2023-05-09

**Authors:** Yangbo Zhang, Wenda Fan, Jiliang Yang, Hao Guan, Qi Zhang, Xi Qin, Changkui Duan, Gabriele G de Boo, Brett C Johnson, Jeffrey C McCallum, Matthew J Sellars, Sven Rogge, Chunming Yin, Jiangfeng Du

**Affiliations:** CAS Key Laboratory of Microscale Magnetic Resonance and School of Physical Sciences, University of Science and Technology of China, Hefei 230026, China; CAS Center for Excellence in Quantum Information and Quantum Physics, University of Science and Technology of China, Hefei 230026, China; CAS Key Laboratory of Microscale Magnetic Resonance and School of Physical Sciences, University of Science and Technology of China, Hefei 230026, China; CAS Center for Excellence in Quantum Information and Quantum Physics, University of Science and Technology of China, Hefei 230026, China; CAS Key Laboratory of Microscale Magnetic Resonance and School of Physical Sciences, University of Science and Technology of China, Hefei 230026, China; CAS Center for Excellence in Quantum Information and Quantum Physics, University of Science and Technology of China, Hefei 230026, China; CAS Key Laboratory of Microscale Magnetic Resonance and School of Physical Sciences, University of Science and Technology of China, Hefei 230026, China; CAS Center for Excellence in Quantum Information and Quantum Physics, University of Science and Technology of China, Hefei 230026, China; Hefei National Laboratory, University of Science and Technology of China, Hefei 230088, China; CAS Key Laboratory of Microscale Magnetic Resonance and School of Physical Sciences, University of Science and Technology of China, Hefei 230026, China; CAS Center for Excellence in Quantum Information and Quantum Physics, University of Science and Technology of China, Hefei 230026, China; CAS Key Laboratory of Microscale Magnetic Resonance and School of Physical Sciences, University of Science and Technology of China, Hefei 230026, China; CAS Center for Excellence in Quantum Information and Quantum Physics, University of Science and Technology of China, Hefei 230026, China; Hefei National Laboratory, University of Science and Technology of China, Hefei 230088, China; CAS Key Laboratory of Microscale Magnetic Resonance and School of Physical Sciences, University of Science and Technology of China, Hefei 230026, China; CAS Center for Excellence in Quantum Information and Quantum Physics, University of Science and Technology of China, Hefei 230026, China; Centre of Excellence for Quantum Computation and Communication Technology, School of Physics, University of New South Wales, NSW 2052, Australia; Centre of Excellence for Quantum Computation and Communication Technology, School of Engineering, RMIT University, Victoria 3001, Australia; Centre of Excellence for Quantum Computation and Communication Technology, School of Physics, University of Melbourne, Victoria 3010, Australia; Centre of Excellence for Quantum Computation and Communication Technology, School of Physics, University of Melbourne, Victoria 3010, Australia; Centre of Excellence for Quantum Computation and Communication Technology, Research School of Physics and Engineering, Australian National University, ACT 0200, Australia; Centre of Excellence for Quantum Computation and Communication Technology, School of Physics, University of New South Wales, NSW 2052, Australia; CAS Key Laboratory of Microscale Magnetic Resonance and School of Physical Sciences, University of Science and Technology of China, Hefei 230026, China; CAS Center for Excellence in Quantum Information and Quantum Physics, University of Science and Technology of China, Hefei 230026, China; Hefei National Laboratory, University of Science and Technology of China, Hefei 230088, China; CAS Key Laboratory of Microscale Magnetic Resonance and School of Physical Sciences, University of Science and Technology of China, Hefei 230026, China; CAS Center for Excellence in Quantum Information and Quantum Physics, University of Science and Technology of China, Hefei 230026, China; Hefei National Laboratory, University of Science and Technology of China, Hefei 230088, China

**Keywords:** single optical centres, erbium ions, RF reflectometry, photoionisation detection

## Abstract

Efficient detection of single optical centres in solids is essential for quantum information processing, sensing and single-photon generation applications. In this work, we use radio-frequency (RF) reflectometry to electrically detect the photoionisation induced by a single Er^3+^ ion in Si. The high bandwidth and sensitivity of the RF reflectometry provide sub-100-ns time resolution for the photoionisation detection. With this technique, the optically excited state lifetime of a single Er^3+^ ion in a Si nano-transistor is measured for the first time to be $0.49 \pm 0.04\ \mu$s. Our results demonstrate an efficient approach for detecting a charge state change induced by Er excitation and relaxation. This approach could be used for fast readout of other single optical centres in solids and is attractive for large-scale integrated optical quantum systems thanks to the multi-channel RF reflectometry demonstrated with frequency multiplexing techniques.

## Teaser

High-bandwidth Radio-frequency reflectometry was used to detect individual photoionisation events, enabling fast detection of single optical centers in solids.

## INTRODUCTION

Efficient detection is key to the discovery and utilisation of single optical centres in solids. Since the first observation on a single-centre basis [1], nitrogen-vacancy (NV) centres in diamond have been used for quantum information processing [2,3], single-photon generation [4] and quantum sensing under ambient conditions [5,6]. The success is built upon strong, spin-dependent fluorescence as well as cyclic optical transitions [7]. Single optical centres possessing similar properties have also been reported in other solids, including common semiconductor materials such as SiC [8–12] and Si [13–15]. More recently, detection and coherent control of single rare-earth ions in solids have been achieved by using cavity coupling [16,17], which is essential to enhance and efficiently collect the photon emission from single rare-earth ions. In these above experiments, the number of collected photons is used as the readout signal. Therefore, the readout fidelity is limited by spin-flip errors, which occur during repeated excitations for getting sufficient signal contrast, and by the photon collection efficiency, despite the improvement due to the use of a solid-immersion lens [18,19].

Recent implementation of spin-to-charge conversion provides spin readout fidelity over 98% for NV centres in diamond [20,21] and divacancy centres in SiC [22]. This is a promising path towards deterministic readout; however, the final charge state readout has a limited speed as it still relies on photon collection within a time window of the order of 1–10 ms. In contrast, electrical charge state readout, widely used in nano-electronic devices, offers high fidelity with short integration times [23,24]. This technique has been demonstrated for single Er^3+^ ions in Si [25–27], but the readout speed in these studies [26,27] was limited by the bandwidth of the current measurement. The readout speed could be significantly increased by adopting radio-frequency (RF) reflectometry [28,29], a fast and sensitive charge detection technique. This technique was first established on Al-based single electron transistors with a bandwidth exceeding 100 MHz [30] and was later applied to semiconductor qubits [31,32]. It is an attractive readout technique for large-scale integrated quantum systems, as it can be performed on devices with only a single electrical contact [33] and also allows multi-channel parallel readout using frequency multiplexing [33,34].

Here, we report photoionisation detection of a single Er^3+^ ion in Si using RF reflectometry. We first introduce the experimental design focusing on the high-bandwidth RF reflectometry technique and then investigate the optically excited state lifetimes of two single Er^3+^ ions. Finally, we analyse the sensitivity and limits of RF detection and discuss future optimisation and other potential applications of this technique.

## RESULTS

**Figure 1.**
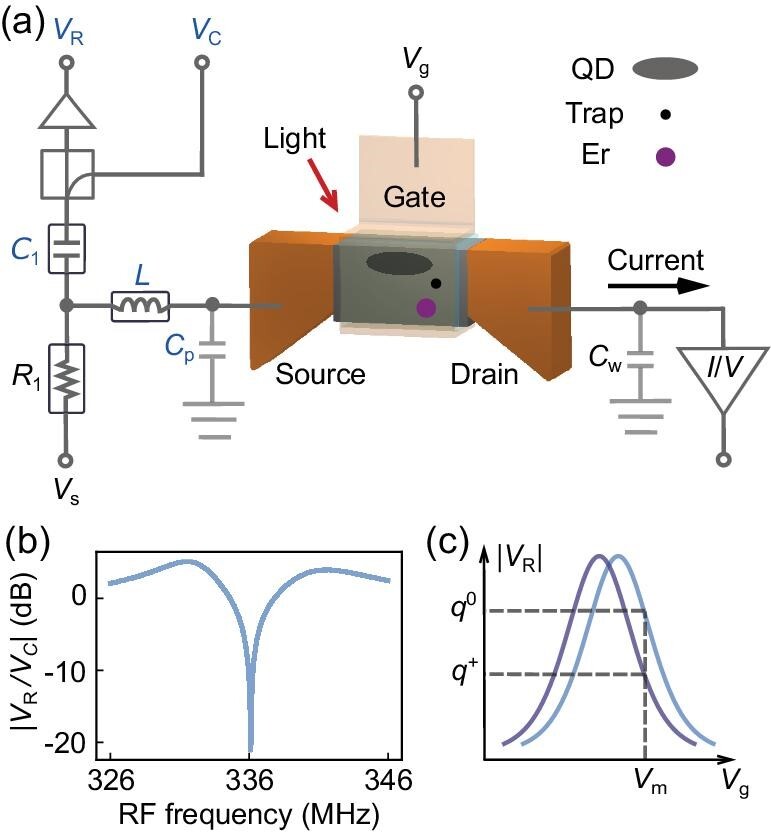
Experimental setup. (a) Circuit diagram showing both RF and DC connections to the device. The DC current is measured from the drain of the device. Its bandwidth is limited by the input impedance of the current pre-amplifier (*I*/*V*), the device resistance and the wiring capacitance *C*_w_. For the RF measurement, a resonant circuit is connected to the source of the device, consisting of a commercial inductor (*L* = 470 nH) and the parasitic capacitance (*C*_p_ = 490.1 fF). A carrier RF signal (*V*_C_) is sent to the resonant circuit, and the reflected signal (*V*_R_) from the resonant circuit can be used to detect an impedance change of the device. A bias tee made up of *R*_1_ and *C*_1_ separates the DC source voltage *V*_s_ and the RF signal so that the DC and RF measurements can be carried out simultaneously. In the FinFET channel, the implanted Er^3+^ ion, a trap and a QD involved in the detection mechanism are displayed. (b) Characteristic of the resonant circuit when all three terminals of the device are grounded. The curve reveals a resonant frequency of 336.1 MHz and a loaded quality factor of 65. (c) Schematic of charge sensing. Loss of an electron, indicated by a change of charge state from *q*^0^ to *q*^+^, induces a shift of the |*V*_R_|-*V*_g_ curve from the light blue curve to the dark blue curve. By choosing a gate voltage on the slope of the curve, *V*_m_, the reflected amplitude at |*V*_R_(*q*^0^)| or |*V*_R_(*q*^+^)| can be used to read out the charge state.

The device used in this work was a Si fin field-effect transistor (FinFET), as illustrated in Fig. 1(a). The FinFET consisted of a crystalline Si nanowire channel (width = 35 nm, length = 130 nm, height = 60 nm) with a dielectric coating, which was surrounded by a polycrystalline Si gate on three sides. The device was placed on a cold stage that was operated at 3.9 K, so single quantum dots (QDs) could form in the channel under sub-threshold gate voltages [35] and work as charge sensors. A small QD filled with a few electrons can detect loss and gain of a single electron occurring more than 100 nm away [36].

The experimental setup had both fibre-optic access for optically exciting single Er^3+^ ions and electrical access for detecting signals from the device, as outlined in Fig. 1(a). Electrical access via twisted-pair and coaxial cables allowed both DC current and RF reflectometry measurements. The DC and RF measurements shared the same electrical connection to the source of the device. However, they could be performed simultaneously without interference, as the low-frequency DC signal and the high-frequency RF signal were separated by a bias tee consisting of a resistor (*R*_1_) and a capacitor (*C*_1_).

The analog bandwidth of the DC measurement was limited to about 2 kHz by the device resistance, the line capacitance of the twisted-pair cables (*C*_w_) and the input impedance of the current pre-amplifier. Therefore, due to the limited bandwidth, the DC measurement was only used for initial device characterisation and as a comparison with the RF signal.

The RF reflectometry measurement relied on a resonant circuit formed of an inductor (*L*) connected to the source of the device and the parasitic capacitance (*C*_p_). A carrier RF signal (*V*_C_) was sent to the resonant circuit, and the reflected RF signal (*V*_R_) was measured. A typical circuit characteristic under the optimal impedance matching condition is shown in Fig. 1(b), revealing a resonant frequency of *f*_r_ = 336.1 MHz and a loaded quality factor of *Q*_r_ = 65. While the inductance and the parasitic capacitance primarily determine the resonant frequency as $f_\mathrm{r} = 1/(2\pi \sqrt{LC_\mathrm{p}})$, the reflected signal is sensitive to small changes in the device impedance thanks to the high quality factor. The resonant circuit’s bandwidth follows *B*_r_ = *f*_r_/*Q*_r_, and this sets an upper limit of 5.2 MHz on the measurement bandwidth. Eventually, a low-pass filter was used to set a bandwidth limit of 2 MHz on the measured RF signal, ensuring a sufficiently low noise level and a fast response. (See the Methods section and Section S1 within the online supplementary material for details on the measurement setup.)

In order to utilise the RF reflectometry for fast charge sensing, the device was operated under a sub-threshold gate voltage so that the device impedance depends on single electron tunnelling through a QD. This results in a sharp peak in the reflected RF signal as a function of the gate voltage, as illustrated by the coloured curves in Fig. 1(c). The peak position is highly sensitive to the electrostatic environment in the vicinity of the QD. When the charge state in the vicinity of the QD changes between *q*^0^ and *q*^+^, the RF signal from the QD switches between the light and the dark blue curves, respectively. In a charge sensing measurement, the gate voltage is typically set on the side of a peak to achieve high sensitivity (e.g. *V*_m_ in Fig. 1(c)), and the measured |*V*_R_| indicates whether the trap is ionised (*q*^+^) or neutral (*q*^0^).

**Figure 2.**
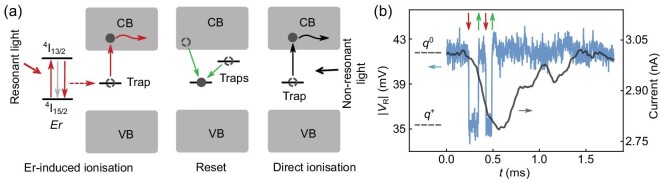
Principle of photoionisation detection of an Er^3+^ ion. (a) Relevant processes of the photoionisation detection of a single Er^3+^ ion. The red arrows represent the processes involved in the Er-induced ionisation, and the green arrows represent the reset of the ionised trap. First, resonant illumination drives an Er^3+^ ion from the ^4^I_15/2_ ground state to the ^4^I_13/2_ optically excited state. Then, due to a non-radiative decay process, an electron bound to a trap is promoted into the conduction band (CB) of Si and subsequently escapes (curved red arrow). The grey arrow represents both the radiative decay processes and other non-radiative decay processes that do not cause the trap to ionise. Finally, the ionised trap can capture an electron from the CB or other localised states (green arrows) and reset to its neutral state. Additionally, non-resonant light can directly ionise the trap (black arrows). Here VB is the valence band of Si. (b) An amplitude-time (|*V*_R_|-*t*) trace (blue) and a current-time trace (black) recorded simultaneously when the device was under resonant CW illumination. The high level in both traces indicates that the trap is in the neutral state (*q*^0^), and the low level indicates that the trap is ionised (*q*^+^). The red and green arrows indicate ionisation and reset events, respectively.

The photoionisation detection involves charge sensing of the ionisation of a single trap following resonant excitation and relaxation of an Er^3+^ ion [27], as illustrated in Fig. 2(a). The Er^3+^ ion can be excited by resonant light from the ^4^I_15/2_ ground state into the ^4^I_13/2_ excited state, and the previous study revealed a single-photon excitation process [27]. Then it decays back to the ground state via either a radiative or non-radiative process. Some non-radiative decay processes can promote an electron bound to a trap into the conduction band, and the electron subsequently escapes. Afterwards, the trap resets to its initial charge state by capturing an electron from a nearby electron reservoir or other localised states. In addition, non-resonant light can also induce direct ionisation of the trap, but this occurs on a much slower time scale than the Er-induced ionisation process and does not display a wavelength dependence. The ionisation and reset events can be detected via charge sensing, as described further below.

Figure 2(b) shows two consecutive pairs of ionisation and reset events indicated by the red and green arrows. The RF and DC signals were recorded simultaneously when the device was under resonant continuous-wave (CW) illumination. In the RF signal, an Er-induced ionisation event (red arrow) induced a signal drop from |*V*_R_(*q*^0^)| to |*V*_R_(*q*^+^)|, and shortly afterwards the signal returned to the original level (|*V*_R_(*q*^0^)|) as the trap reset stochastically (green arrow). In comparison, the DC current also shows a response to the four events consistent with the RF signal, but the bandwidth-limited features in the DC signal prohibit accurate identification of these events. In addition, the RF signal can pick up ionisation events that reset too rapidly to be recognized from the DC signal. By scanning the laser frequency and detecting the ionisation events, two Er^3+^ transitions were identified in this device. They can be selectively excited using their specific resonant frequencies. The zero-field frequency is 195 054.0 GHz (1536.972 nm) for Er1, and 195 940.7 GHz (1530.016 nm) for Er2. See Section S3 within the online supplementary material for the Zeeman splitting spectra of the two Er^3+^ transitions.

**Figure 3.**
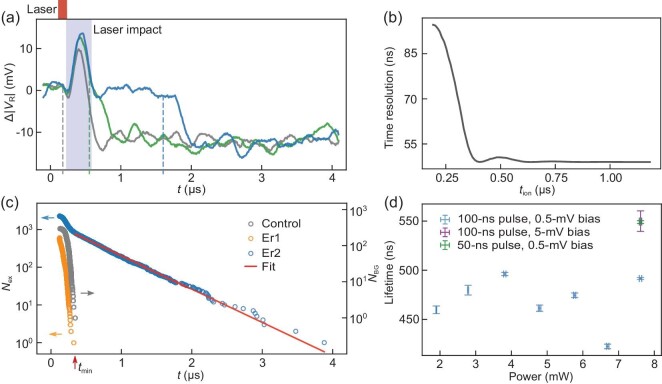
Lifetime measurement. (a) (Upper) Typical experimental sequence. In each measurement cycle, a laser pulse (red) is applied for resonant excitation. (Lower) Three typical Δ|*V*_R_|-*t* traces measured from Er2. Here, a relative amplitude Δ|*V*_R_| is calculated by subtracting the averaged value of a 5-*μ*s time trace prior to the laser pulse from the measured amplitude |*V*_R_|. The region shaded in light blue corresponds a ‘laser impact’ time window in which a laser-induced transient jump appears. The ionisation time *t*_ion_ is determined by modelling the laser impact and the ionisation event, and the dashed lines indicate the fitted ionisation time, *t*_ion_, which is 0.18 *μ*s for the grey trace, 0.55 *μ*s for the green trace and 1.60 *μ*s for the blue trace. (b) Time resolution of the photoionisation detection as a function of *t*_ion_. The small hump at 0.5 *μ*s is caused by the low-pass filtering. (c) Statistics of ionisation events measured from Er1 (orange), Er2 (blue) and background ionisation events (grey). Here *N*_ex_(*t*) and *N*_BG_(*t*) represent the number of cycles in which the trap remains neutral at time *t* among all observed photoionisation cycles. An exponential fit (red line) to selected Er2 events (*t*_ion_ > *t*_min_) gives an excited state lifetime of τ_ex_ = 492 ± 1 ns for Er2. (d) Excited state lifetime τ_ex_ of Er2 measured at different laser powers (blue), a different pulse length (green) and a different source-drain bias voltage (purple). No significant impact on τ_ex_ is observed. The *x*- and *y*-axis error bars represent the uncertainty of laser power and the fitting error of τ_ex_, respectively.

The photoionisation detection can also be carried out with pulsed laser excitation. Figure 3(a) shows three typical traces that contain an ionisation event. A 7.6 mW–100 ns laser pulse (upper panel of Fig. 3(a)) was applied in each measurement cycle. The appearance of a low-level (−12 mV) signal indicates a trap ionisation event in the measurement cycle, similar to the RF signal response observed in the CW measurement as shown in Fig. 2(b). In addition, the laser pulse causes a transient signal jump at $t = {0.4}\,\, \mu\mathrm{s}$ in the ‘laser impact’ time window (shaded in light blue in Fig. 3(a)). This phenomenon occurs for all measurement cycles regardless of the laser frequency. The transient jump becomes more significant for longer laser pulses and/or higher laser powers. To maintain a high detection sensitivity, we kept the maximum laser power and pulse length at 7.6 mW and 100 ns, respectively.

In order to determine the ionisation time, *t*_ion_, we use a model that includes the laser-induced transient jump, the ionisation-induced signal drop and low-pass filtering. The dashed lines in Fig. 3(a) indicate the fitted ionisation times for the three traces. When an ionisation event occurs after the ‘laser impact’ time window, a well-isolated signal drop can be observed, for example, between 1.8 and 2.0 *μ*s on the blue trace. In contrast, the ionisation-induced signal drop on the green curve slightly overlaps with the falling edge of the transient jump as the ionisation event occurred shortly after the laser pulse, while the ionisation-induced signal drop on the grey curve merges with the transient jump as the ionisation event occurred during the laser pulse.

We then analyse the time resolution of the photoionisation detection. The noise in the RF signal and the fluctuation of the ionisation-induced signal drop directly affect the stability of the ionisation-induced falling edge and, thus, the ionisation time. These two factors contribute to 56-ns time resolution. Additionally, the laser-induced transient jump can influence the fitting accuracy of the ionisation time for events occurring during or shortly after the laser pulse. By considering all the above effects, the time resolution of the photoionisation detection is analysed and plotted as a function of ionisation time, *t*_ion_, in Fig. 3(b). Details of fitting and simulation can be found in Section S6 within the online supplementary material.

To investigate the excited state lifetime, pulsed detection was performed for Er1 and Er2 with 7.6 mW–100 ns resonant laser pulses. The short excitation pulse resulted in relatively low ionisation probabilities of ∼6 × 10^−4^ and ∼3 × 10^−4^ events per measurement cycle for Er1 and Er2 and 594 and 2280 ionisation events were recorded, respectively. These events include Er-induced ionisation events and frequency-independent background events. A control experiment was carried out with 7.6-mW–100-ns non-resonant laser pulses, and 294 background events were recorded at an ionisation probability of ∼6 × 10^−5^ events per cycle.

In the pulsed detection, the Er^3+^ ion can be excited into the ^4^I_13/2_ excited state by a laser pulse and then relax back to the ^4^I_15/2_ ground state with a certain probability of inducing an ionisation event. The statistics of the Er-induced ionisation events should follow an exponential decay function: $N_{\mathrm{ex}}(t) = N_{\mathrm{ex}}(t_0) \times e^{(t_0-t)/\tau _{\mathrm{ex}}}$. Here *N*_ex_(*t*) is the number of cycles in which the Er^3+^ ion is in the excited state at time *t* and τ_ex_ is the excited state lifetime.

Statistics of the ionisation events from the three experiments are shown in Fig. 3(c). All the ionisation events from the control experiment (grey) occurred during or shortly after the laser pulse. Therefore, the latest ionisation time in the control experiment can be used as a minimum threshold (*t*_min_ = 343 ns) to exclude the background events. As shown in Fig. 3(c), 819 ionisation events occurring after *t*_min_ are selected from the Er2 measurement (blue), and an exponential decay fit (red line) to these events gives an excited state lifetime of Er2, τ_ex_ = 492(1) ns. This threshold, *t*_min_, is also used to exclude background events for generating the Zeeman splitting spectrum of Er2 (Fig. S3b within the online supplementary material). In contrast, all the ionisation events from the Er1 measurement (orange points in Fig. 3(c)) occurred before *t*_min_. As a result, the background events cannot be excluded using the selection method. Nevertheless, we can estimate the background count in the Er1 measurement based on the ionisation probability in the control experiment. Approximately, 90% of the 594 events are expected to be Er-induced ionisation events. We infer that the excited state lifetime of Er1 is shorter than 50 ns, but cannot determine it accurately due to the limited time resolution of the detection for these events.

We then investigated the effects of laser power, pulse length and source-drain bias voltage on the excited state lifetime of Er2. These factors may influence the local environment in the channel of the device, such as the amount of photo-induced carriers and the electric field, which may affect the excited state lifetime of an Er^3+^ ion. We first varied the laser power from 1.9 to 7.6 mW at a pulse length of 100 ns and a bias voltage of *V*_s_ = 0.5 mV, and the results are plotted as blue markers in Fig. 3(d). While keeping the laser power fixed at 7.6 mW, we shortened the pulse length from 100 to 50 ns in one experiment (green marker) with *V*_s_ = 0.5 mV and increased the bias voltage to *V*_s_ = 5.0 mV in another experiment (purple marker) with a pulse length of 100 ns. Overall, the laser power, the pulse length or the source-drain bias voltage does not affect the excited state lifetime of Er2 significantly, as shown in Fig. 3(d). See Section S8 within the online supplementary material for detailed experimental and fitting results.

In the final section, we analysed the sensitivity and limits of the RF detection, including the signal-to-noise ratio (SNR) of the RF reflectometry and fidelity of the photoionisation detection, which are vital to enable the fast detection of optical centres.

**Figure 4.**
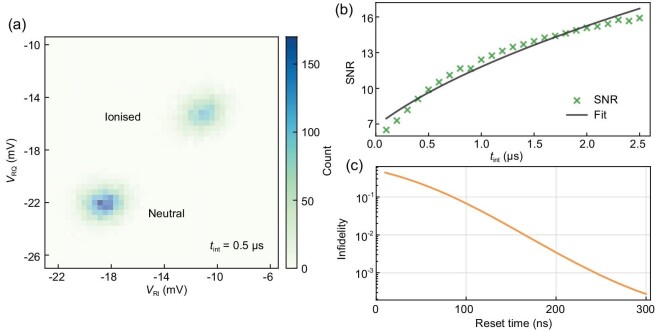
Signal-to-noise ratio (SNR) and detection fidelity. (a) A 2D histogram of the reflected RF signal *V*_R_ in the *IQ* plane with an integration time of 0.5 *μ*s. Here *V*_RI_ and *V*_RQ_ stand for the in-phase and quadrature components of *V*_R_, respectively. Count is the number of data points (*V*_RI_, *V*_RQ_) appearing in each histogram bin. Two well-separated regions correspond to the ionised and neutral states of the trap. (b) SNR as a function of the integration time. The black curve is a fit with $\mathrm{SNR}=\mathrm{SNR}_{{1}\, {\mu\mathrm{s}}} \times \sqrt{(t_0+t_{\mathrm{int}})/ {1}\,\, {\mu\mathrm{s}}}$, where $\mathrm{SNR}_{{1}\, {\mu\mathrm{s}}}$ is a characteristic SNR with an effective integration time of 1 *μ*s and *t*_0_ is an intrinsic integration time. (c) Infidelity of the photoionisation detection as a function of the reset time.

In the present study, the reflected RF signal, *V*_R_, is mainly sensitive to changes in the device resistance, which is typical when the resonant circuit is connected to the source or drain. In general, a change of charge state can induce both resistive and reactive responses, which lead to changes in the amplitude and phase of *V*_R_. To fully characterise the SNR, the in-phase and quadrature components of *V*_R_ are plotted as a two-dimensional (2D) histogram in the *IQ* plane. Figure 4(a) shows a 2D histogram of the reflected RF signals before and after an ionisation event with a signal integration time of *t*_int_ = 0.5 *μ*s. Two well-separated regions correspond to the ionised and neutral states of the trap. The SNR is calculated as SNR = |Δ*V*_R_|/σ_*V*_, where |Δ*V*_R_| is the centre-to-centre distance between the two regions and σ_*V*_ is their averaged standard deviation. The SNR values calculated with different integration times are shown in Fig. 4(b). This dependence can be described by the equation $\mathrm{SNR}=\mathrm{SNR}_{{1}\, {\mu\mathrm{s}}} \times \sqrt{(t_0+t_{\mathrm{int}})/ {1}\,\, {\mu\mathrm{s}}}$ [37], and the fit (black line in Fig. 4(b)) yields $\mathrm{SNR}_{{1}\, {\mu\mathrm{s}}} = 9.6$, a characteristic SNR for an effective integration time of 1 *μ*s, and $t_0 = {0.50(6)}\,\, {\mu\mathrm{s}}$, an intrinsic integration time corresponding to the measurement bandwidth of 2 MHz.

The fidelity of the photoionisation detection is influenced by the SNR of the RF reflectometry and the duration of the ionisation signal, equivalent to the reset time. In the present device, the SNR with any integration time above 0.5 *μ*s exceeds 10, and the reset time constant is 70.9 ± 0.1 *μ*s. As a result, most ionisation events can be detected with near-unity fidelity. Nevertheless, 0.7% of all ionisation events are expected to reset within 0.5 *μ*s at the limit of the detection bandwidth. These short-lived events would have attenuated ionisation signal contrast and, consequently, could be missed in the detection. In order to quantify this impact, we simulated the infidelity of photoionisation detection for short-lived ionisation events, as shown in Fig. 4(c). For each reset time, the infidelity is calculated using the expected signal amplitude and the measured noise. (See Section S7 within the online supplementary material for details.) The simulation results reveal 99% detection fidelity for ionisation events with a duration longer than 170 ns, and overall detection fidelity of 99.96% for all ionisation events in the present device.

## DISCUSSION

We have demonstrated fast photoionisation detection of single Er^3+^ ions in a Si nano-transistor using RF reflectometry. At an analog bandwidth of 2 MHz, this technique provides a SNR of 9.6 with an integration time of 0.5 *μ*s and a photoionisation detection time resolution below 100 ns. The fast detection enables ^4^I_13/2_ excited state lifetime measurements for single Er^3+^ ions in a Si nano-transistor.

The SNR and time resolution of RF readout can be further improved. Firstly, a lower-noise first-stage amplifier could further reduce the noise level of the measured RF signal, for example, a superconducting quantum interference device amplifier [38] or a Josephson parametric amplifier [39,40]. Secondly, replacing the surface mount inductor with a superconducting inductor [41] can minimise dissipative losses and reduce the parasitic capacitance, enhancing the signal contrast. However, using a superconducting inductor constrains high magnetic fields to be in the plane of the superconducting film, which should be considered in device design. As an example, robust charge sensitivity has been demonstrated in moderate in-plane magnetic fields up to 1 T [41]. Finally, part of the signal fluctuations on a time scale of microseconds are likely due to light-induced heating and random photocarrier generation in the leads of the FinFET device, as a large area of the leads around the channel was illuminated by the divergent light in the present study. This impact can be reduced in new device structures with an Er-doped region separated from the channel. Also, undesirable illumination and laser heating can be further reduced by coupling the Er-doped region to an optical cavity or waveguide [42].

The above SNR improvements would also provide larger signal margins for increasing the detection bandwidth. The physical bandwidth of RF reflectometry is ultimately limited by *f*_r_/*Q*_r_ and can be increased by building a resonant circuit with a higher resonance frequency *f*_r_ and/or by lowering the quality factor *Q*_r_. However, a lower quality factor leads to lower detection sensitivity [29], and detection at a higher physical bandwidth is susceptible to higher frequency noises. Therefore, the trade-off between sensitivity and bandwidth must be assessed and higher frequency noise suppression should be investigated in order to utilise the higher bandwidth.

RF reflectometry provides a promising direction forward for scalable optical quantum systems, and it is compatible with the microwave drive for spin manipulation as demonstrated in quantum electronic devices [43,44]. The photoionisation detection of single Er^3+^ ions relied on Er-induced ionisation of a nearby trap; however, the charge state change observed from NV centres in diamond [20,21] and single defects in SiC [22] could be directly detected by a charge sensor without involving an additional trap. The RF resonant circuit was connected to the source of the device in the present study; as a result, a dissipative response, i.e. the reflected amplitude, was used for detection. Alternatively, the resonant circuit can be connected to the gate for dispersive readout, which has been implemented on single lead QD devices [33]. This structural simplification, combined with channel multiplexing techniques [45], provides an attractive solution to the readout and control of single optical centres in large-scale integrated systems.

## METHODS

The device was mounted on a printed circuit board (PCB) and placed on a cold stage that was operated at 3.9 K in a liquid-helium-free cryogenic system. For the RF measurement, a carrier signal from an RF signal generator was split into two parts, one sent to the device and the other used for signal demodulation. The first part passed through room-temperature (RT) attenuators (−36 dB), cryogenic attenuators (−40 dB) and a directional coupler and then reached the device PCB. The reflected RF signal went through the directional coupler via a different path from the incoming RF signal and was then amplified by a commercial cryogenic amplifier, a homemade cryogenic amplifier and an RT amplifier sequentially. The amplified signal and the second part of the original RF signal were sent to a quadrature demodulator for homodyne demodulation. Finally, the demodulated signals, *V*_RI_ and *V*_RQ_, were sent to an oscilloscope after passing a 2-MHz low-pass filter.

The laser pulses were generated by an acousto-optic modulator that was controlled by a pulse generator. A small portion of the pulsed light was sent to a photodetector, and the output voltage was sent to the oscilloscope for triggering and power monitoring. Specifically, the rising edge of the laser pulse captured by the photodetector was used as the trigger in the present study and corresponds to *t* = 0. Because of instrumental response times and latencies of optical fibres and coaxial cables, the arrival time of the laser pulse at the device is estimated to be *t* = 30 ns. The induced RF signal at this specific moment should start to appear in the oscilloscope signal at *t* = 75 ns, but actually shows up much later (about $t=0.4\,\, \mu\mathrm{s}$ in Fig. 3(a)) due to the 2-MHz low-pass filtering. These time delays remained stable within ±1 ns and should not affect the analysis of lifetime and time resolution, as we did not change the lengths of fibres and cables throughout the present study. More details about the connections can be found in Section S1 within the online supplementary material.

## Supplementary Material

nwad134_Supplemental_File https://doi.org/10.57760/sciencedb.08209
