# Supplementary material for: Photoionisation detection of a single Er3+ ion with sub-100-ns time resolution
Source: Natl Sci Rev. 2023 May 9;11(4):nwad134. doi: 10.1093/nsr/nwad134 (PMC10939366; doi:10.1093/nsr/nwad134)
Supplement: nwad134_Supplemental_File — https://doi.org/10.57760/sciencedb.08209 [file nwad134_supplemental_file.pdf]

Supplementary material for ‘Photoionisation detection of a single erbium ion with sub-100-ns time resolution’

Yangbo Zhang,[1,3] Wenda Fan,[1,3] Jiliang Yang,[1,3] Hao Guan,[1,2,3] Qi Zhang,[1,3]
Xi Qin,[1,2,3] Changkui Duan,[1,3] Gabriele G. de Boo,[4] Brett C. Johnson,[5,6]
Jeffrey C. McCallum,[6] Matthew J. Sellars,[7] Sven Rogge,[4]
Chunming Yin,[1,2,3,*] Jiangfeng Du[1,2,3]

[1]CAS Key Laboratory of Microscale Magnetic Resonance and School of Physical Sciences, University of Science and Technology of China, Hefei 230026, China,
[2]Hefei National Laboratory, University of Science and Technology of China, Hefei 230088, China,
[3]CAS Center for Excellence in Quantum Information and Quantum Physics, University of Science and Technology of China, Hefei 230026, China,
[4]Centre of Excellence for Quantum Computation and Communication Technology, School of Physics, University of New South Wales, NSW 2052, Australia,
[5]Centre of Excellence for Quantum Computation and Communication Technology, School of Engineering, RMIT University, Victoria 3001, Australia,
[6]Centre of Excellence for Quantum Computation and Communication Technology, School of Physics, University of Melbourne, Victoria 3010, Australia,
[7]Centre of Excellence for Quantum Computation and Communication Technology, Research School of Physics and Engineering, Australian National University, ACT 0200, Australia,

***Corresponding author.** Email: Chunming@ustc.edu.cn

I. MEASUREMENT SETUP

The measurement setup had both fibre-optic access for optically exciting single Er^{3+} ions and electrical access for detecting signals from the device, as shown in Fig. 1.

For optical access, laser light from a wavelength-tunable laser (Pure Photonics PPCL550) passed through an acousto-optical modulator (G&H T-M200-0.1C2J-3-F2S), and was then divided to two paths by a beam splitter (Thorlabs TW1550R2A2). 10% of the laser light was sent to a photodetector (Thorlabs PDA05CF2) for power monitoring and triggering, and the rest was sent into a closed-cycle cryostat (Bluefors BF-LD250) to illuminate the FinFET device with a divergent beam.

The electrical access allowed both DC current and RF measurements. A DC voltage source (Stahl-Electronics BS 1-16) was use to bias the source and gate of the FinFET, and a reverse-biased GaAs varactor (C_{var}) to optimise the impedance matching to the FinFET [44]. DC current from drain of FinFET was amplified by a low-noise current pre-amplifier (SRS SR570) and then was recorded by an oscilloscope (Tektronix MDO3104).

For the RF measurement, a carrier tone from a RF signal generator (SRS SG386) was first divided into two parts by a power splitter (Mini-circuits ZFRSC-183-S+). One part was sent to a quadrature demodulator (Mini-circuits AD0105B) as a demodulation reference signal. The other part went through several attenuators and a directional coupler before reaching the RF port of the device PCB. The reflected RF signal first went through the directional coupler, and then was amplified by a commercial cryogenic amplifier (Low Noise Factory LNF-LNC0.2_3A), a homemade cryogenic amplifier and a RT amplifier (Mini-circuits ZX60-P103LN+), and finally reached the quadrature demodulator. The demodulated V_{RI} and V_{RQ} signals were recorded by the oscilloscope after passing through a homemade low-pass filter. The cutoff frequency of the low-pass filter was set to 2 MHz.

II. RF AND DC COULOMB PEAKS

Figure 2 shows two gate-voltage dependence traces measured simultaneously in DC current and RF reflection ($|V_{\mathrm{R}}|$) under a source-drain bias voltage of 0.5 mV. The two traces present almost identical features, which indicates that the DC and RF measurements probe the same quantum states. In addition, multiple overlapping Coulomb peaks appear in the traces, and correspond to quantum tunnelling from multiple quantum states. To achieve a high sensitivity, the gate voltage was set on the slope ($V_{\mathrm{m}} = 578.0$ mV) for the photoionisation detection.

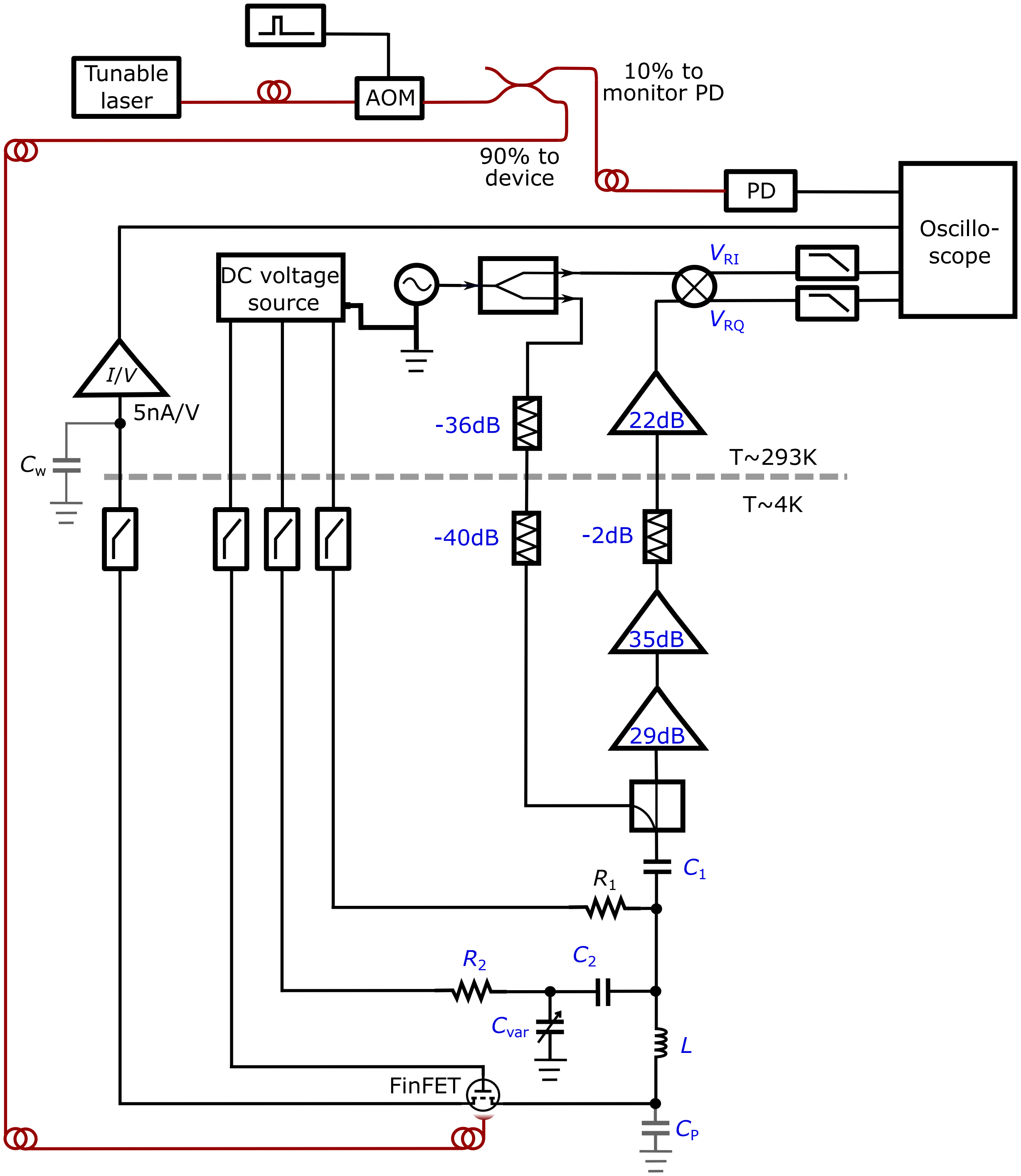

FIG. 1. **A schematic of the measurement setup.** Dark red lines represent optical fibres and fibre components, and black lines represent the electrical connections.

III. SPECTRA OF THE TWO Er^{3+} TRANSITIONS

Figures 3(a,b) show the Zeeman splitting spectra of the two Er^{3+} transitions, named Er1 and Er2. They can be selectively excited using their specific resonant frequencies. The zero-field frequency is 195 054.0 GHz (1536.972 nm) for Er1, and 195 940.7 GHz (1530.016 ns) for Er2.

The Zeeman splitting spectrum of Er1 in Fig. 3(a) was measured under CW illumination. A $|V_{\mathrm{R}}|$-t trace was recorded for 40 ms at each laser frequency, as the laser frequency scanned across the resonance in one applied magnetic field. This

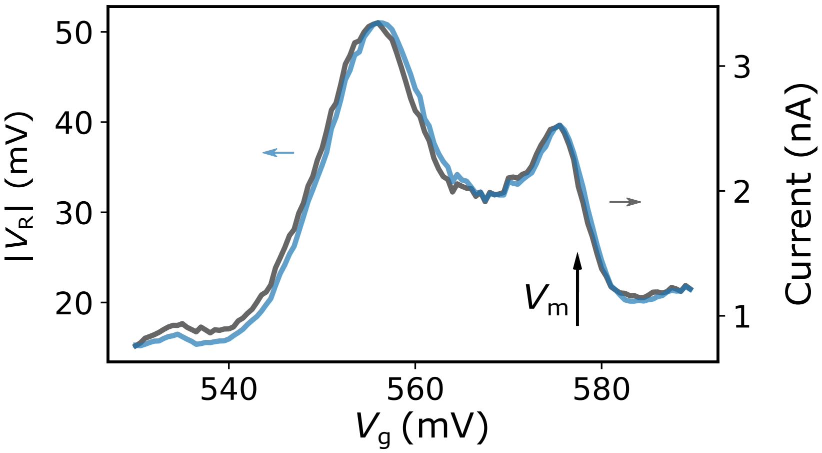

FIG. 2. **RF and DC Coulomb peaks.** Coulomb peaks measured simultaneously in DC current and RF reflection. The gate voltage is set on the slope of the traces, $V_m = 578.0$ mV, for the photoionisation detection.

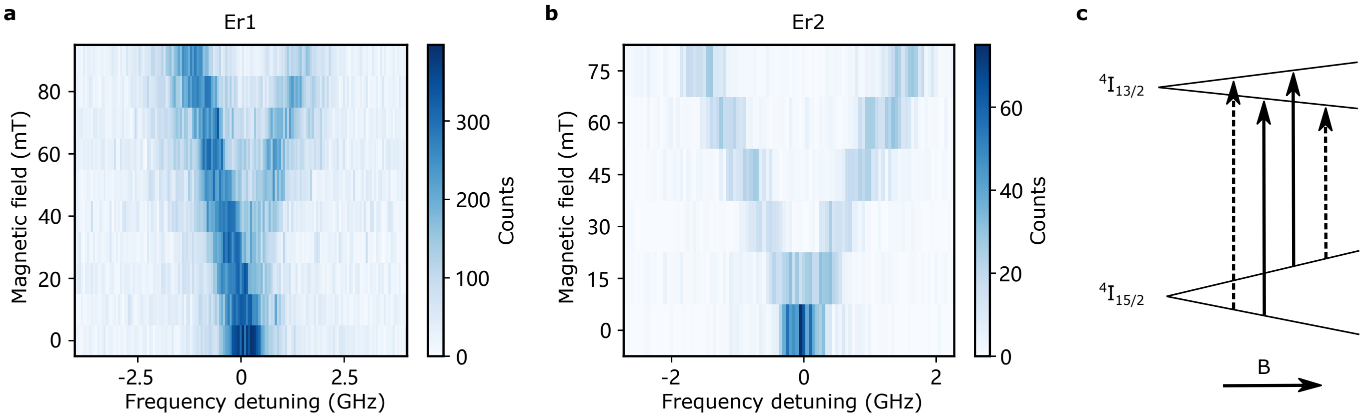

FIG. 3. **Zeeman splitting spectra of the two Er^{3+} transitions. a**, Zeeman splitting spectrum of Er1 with a centre frequency of 195 054.0 GHz. Each pixel corresponds a 40 ms $|V_R|$-t trace measured under 556 µW CW illumination, and the colour represents the number of photoionisation events in one trace. **b**, Zeeman splitting spectrum of Er2 with a centre frequency of 195 940.7 GHz. Each pixel corresponds to 100 000 repeated pulsed measurements with a pulse length of 300 ns and a power of 7.6 mW. The colour represents the number of selected Er-induced events with the selection method described in the main text. **c**, Energy level diagram showing the Zeeman splitting and optical transitions of an Er^{3+} ions in Si.

measurement was then repeated in several magnetic fields, and the ionisation events in each trace were counted. Some events are induced by the direct ionisation (main figure 2a) and contribute to a wavelength-independent background count of about 50 in each trace, whereas the other events are induced by Er excitation and relaxation. The total count in each trace is then plotted as a function of laser frequency detuning and magnetic field in Fig. 3(a).

In comparison to Er1, Er2 has a lower count rate, which is comparable to the background count rate. This leads to a low spectral contrast under CW illumination. In order to enhance the contrast, pulsed detection and post-selection are used to identify only Er-induced ionisation events, as presented in the next section. The selected count is plotted as a function of laser frequency detuning and magnetic field in Fig. 3(b).

In a magnetic field, both the $^4I_{15/2}$ ground and $^4I_{13/2}$ excited states split into two electron spin levels, and four optical transitions can be observed, as shown in Fig. 3(c). Typically, the two spin-conserving transitions, indicated by the solid-line arrows, appear stronger than the two spin-flip transitions, indicated by the dashed-line arrows. We conclude that the two Zeeman arms in Figs. 3(a,b) correspond to the spin-conserving transitions.

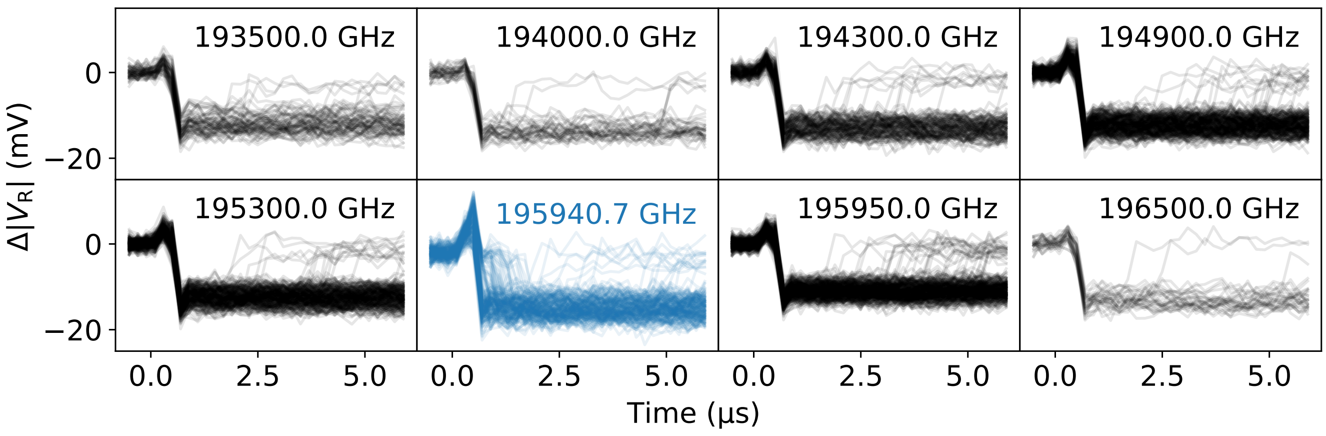

FIG. 4. **Comparison of photoionisation events under resonant and the non-resonant illumination.** Time traces from non-resonant control measurements (black) and an Er2 photoionisation measurement (blue). The laser power and pulse length are 7.6 mW and 100 ns, respectively. The laser frequency is marked in each panel.

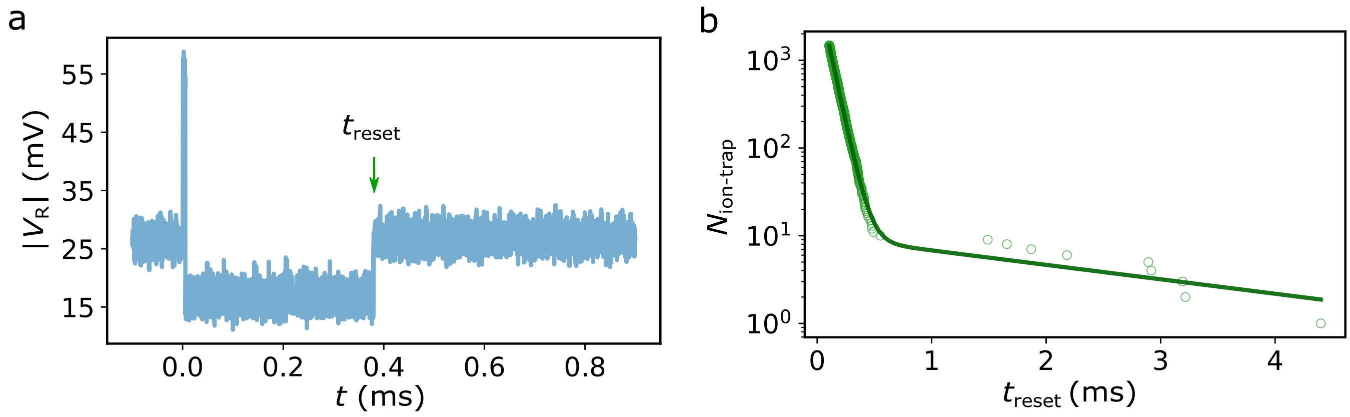

FIG. 5. **Trap reset time measurement.** **a**, A reflected RF amplitude $|V_R|$-time trace with the complete ionisation-reset events measured when a 5 μs resonant laser pulse illuminate device. The reset time t_{reset} can be easily determined from the $|V_R|$-time trace. **b**, The statistic of reset time t_{reset} from 1480 reset events. $N_{ion-trap}$ represents the counts of ionised trap at the observation time. Through fitting $N_{ion-trap}$ to a double exponential decay, the fast and slow decay time of ionised trap are 70.9 ± 0.1 μs and 2.64 ± 0.07 ms, respectively.

IV. COMPARISON OF PHOTOIONISATION EVENTS UNDER RESONANT AND THE NON-RESONANT ILLUMINATION

As shown in Fig. 3c in the main text, the photoionisation events induced by non-resonant laser pulses occurred within 343 ns since the start of the laser pulse, but a number of events occurred much later than 343 ns when Er2 was under resonant excitation. The difference in ionisation times is evident in the time traces shown in Fig. 4. The black curves were measured with several different laser frequencies, and all ionisation events happened shortly after the laser pulse and led to closely overlapping fall edges. In contrast, the blue curves were from the Er2 photoionisation measurement, and a number of delayed falling edges can be observed between $t = 1$ μs and 2 μs.

V. TRAP RESET TIME MEASUREMENT

To determine how fast the trap resets after an ionisation event, a long readout time measurement was carried out. In each measurement cycle, a 5 μs laser pulse resonant with Er1 was applied and a 10 ms long time trace was recorded. Figure 5(a) shows a typical $|V_R|$-time trace, and the trap reset time is indicated by the green arrow. In total, 1480 ionisation events were measured, and the statistics of the reset times is shown in Fig. 5(b). While the majority of the events reset within 1 ms, a few events reset at a later time. A double exponential decay fit gives a fast decay time of

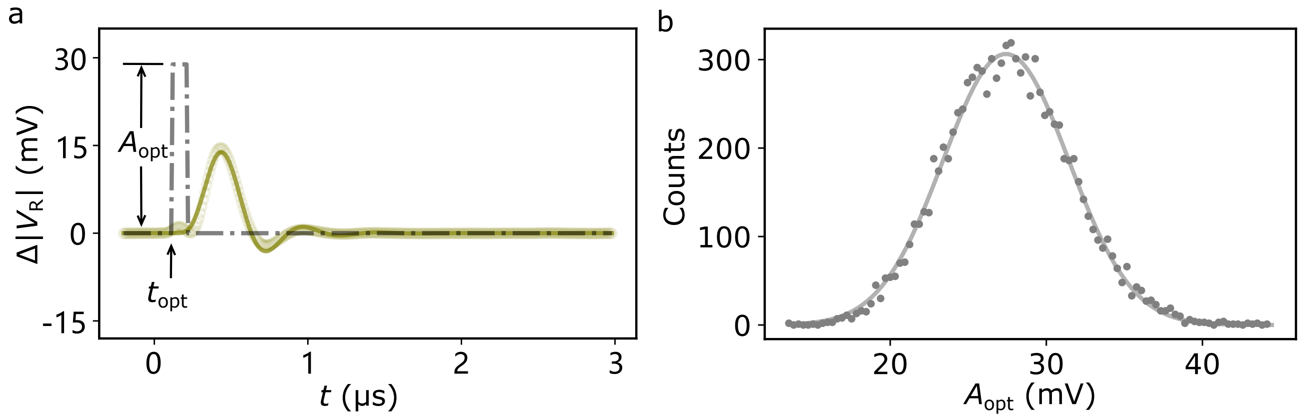

FIG. 6. **Simulation of the laser-induced impact.** **a**, An averaged trace (yellow circle) from 2500 idle cycles showing the laser-induced impact. The dash-dotted grey line shows the RF signal at the device without considering any bandwidth limitation, while the yellow line is final simulation result with the 2 MHz low-pass filtering. Here, $t_{\rm opt}$ represents the start time of the laser-induced impact, respectively, and $A_{\rm opt}$ is the amplitude of the laser-induced impact on the RF signal. **b**, A histogram (dot) of $A_{\rm opt}$ from the 10 000 idle cycles fitted with a Gaussian function (line).

$70.9 \pm 0.1\,\mu$s and a slow decay time of $2.64 \pm 0.07\,$ms.

VI. FITTING OF THE IONISATION TIME

Accurate determination of ionisation time $t_{\rm ion}$ is essential for investigating dynamic processes, such as the $^4{\rm I}_{13/2}$ excited state lifetime. We first use the idle cycles in which no ionisation event occurs to determine the parameters associated with the laser-induced impact, and then fit the ionisation events.

Figure 6(a) shows an averaged $|V_{\rm R}|$-time trace (yellow circle) from 2500 idle cycles. The laser-induced impact includes the peak at approximately $t = 0.5\,\mu$s and the ringing afterwards. We first consider the RF signal at the device and then apply low-pass filtering to simulate the measured signal. A rectangular pulse (dash-dotted grey line) is used to model the RF signal at the device. The pulse length equals the laser pulse length, and the fitting parameters are the start time ($t_{\rm opt}$) and the amplitude ($A_{\rm opt}$) of laser-induced pulse. A simulated 2 MHz low-pass filtering is then applied to the rectangular pulse. The final fitting result is plotted as the yellow line in Fig. 6(a) and gives $t_{\rm opt} = 122\,$ns and $A_{\rm opt} = 28.9\,$mV.

Next, the ionisation events are fitted on an individual basis. In each cycle, the laser-induced rectangular pulse and an ionisation-induced falling edge are considered, as shown by the dash-dotted lines in Figs. 7(a,b). The two parameters of the rectangular pulse, $t_{\rm opt}$ and $A_{\rm opt}$, determined from the idle cycles (Fig. 6(a)), are directly applied here. For the ionisation-induced falling edge, the amplitude of the falling edge, $A_{\rm Er}$, is calculated as the difference between the time-averaged signal before the laser pulse and after the falling edge in each cycle, while the ionisation time, $t_{\rm ion}$, is treated as a free parameter in the fitting of an ionisation event.

Two typical ionisation events are shown in Figs. 7(a,b). The blue circles represent the measured $\Delta|V_{\rm R}|$-time traces. The amplitudes of the falling edges calculated from the traces in Figs. 7(a,b) are $A_{\rm Er} = -9.9\,$mV and $A_{\rm Er} = -11.3\,$mV, respectively. The fitting results are plotted as the solid blue lines in Fig. 7(a,b) and give the ionisation times, $t_{\rm ion} = 0.27\,\mu$s and $t_{\rm ion} = 0.92\,\mu$s, respectively.

Last, we analyse the resolution of the fitted ionisation time. The analysis consists of two parts, a timing jitter of the ionisation-induced falling edge and the fitting uncertainties due to the laser-induced transient jump.

The noise in the RF signal and the amplitude fluctuation of the ionisation-induced falling edge directly affect the stability of the ionisation-induced falling edge and lead to a timing jitter of the ionisation time. Figure 7(c) shows multiple traces plotted as a function of $(t - t_{\rm ion})$, a relative time with respect to the ionisation time. The noise in the RF signal, $\sigma_{\rm noise}$, is calculated as the standard deviation extracted from the histogram of $\Delta|V_{\rm R}|$ before the laser pulse. The amplitude fluctuation of the ionisation-induced falling edge, $\sigma_{\rm Er}$, is calculated as the standard deviation in the distribution of $A_{\rm Er}$. Both factors lead to a timing jitter in the occurrence time of the ionisation-induced falling edge, and the measure of timing jitter is the width of the statistical distribution in these occurrence times [45]. The

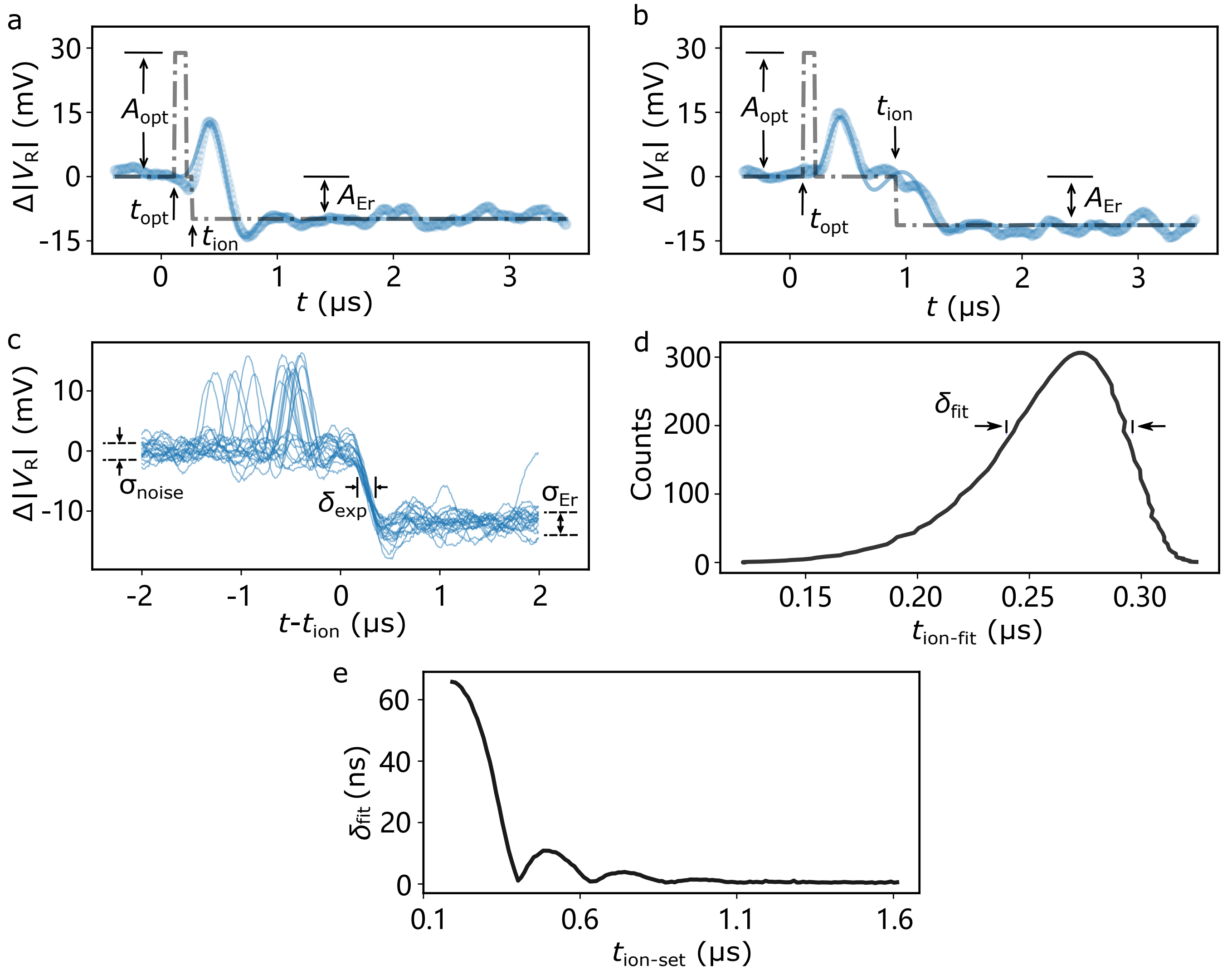

FIG. 7. **Determination of the ionisation time.** **a** and **b**, Measured and fitted traces from two typical ionisation events. The blue circles represent the measured $\Delta|V_R|$-time traces. The dash-dotted grey lines are the RF signal at the device without considering any bandwidth limitation, while the blue lines are final simulation result with the 2 MHz low-pass filtering. Here, t_{ion} represents the Er-induced ionisation time, and A_{Er} is the amplitude of the ionisation-induced falling edge. **c**, Multiple ionisation traces plotted as a function of $t - t_{ion}$, a relative time with respect to the ionisation time. Here, δ_{exp} stands for the timing jitter of the ionisation-induced falling edge, σ_{noise} represents the noise in the RF signal, and σ_{Er} represents the fluctuation of A_{Er}. **d**, Distribution of the fitted ionisation time due to the variation of A_{opt} for a set ionisation time, (272 ns). The standard deviation, $\delta_{fit} = 28.1$ ns, corresponds to the uncertainty of the fitted ionisation time. **e**, Uncertainty of the fitted ionisation time as a function of the set ionisation time. The fluctuation between 0.4 μs and 0.9 μs is caused by the low-pass filtering.

timing jitter can be calculated as:

$$\delta_{exp} = \sqrt{(\sigma_{noise}^2 + \sigma_{Er}^2)/\alpha^2 + \delta_{instr}^2},$$

where α is the slope of the ionisation induced falling edge, and δ_{instr} is the instrumental contribution to timing jitter, which is about 1.5 ns in this study. Overall, the timing jitter of is 28 ns.

The second part of the analysis focuses on the impact of laser-induced transient jump on the fitting of the ionisation time. This impact is significant for the ionisation events that occur during or shortly after the laser pulse, such as the grey and green traces in Fig. 3a in the main text and the trace in Fig. 7(a). The two parameters of the rectangular pulse, t_{opt} and A_{opt}, determined from the idle cycles (Fig. 6(a)), are directly applied in the fitting of the ionisation time, however, A_{opt} does fluctuate as shown by the distribution of A_{opt} in Fig. 6(b). In contrast, the laser-induced

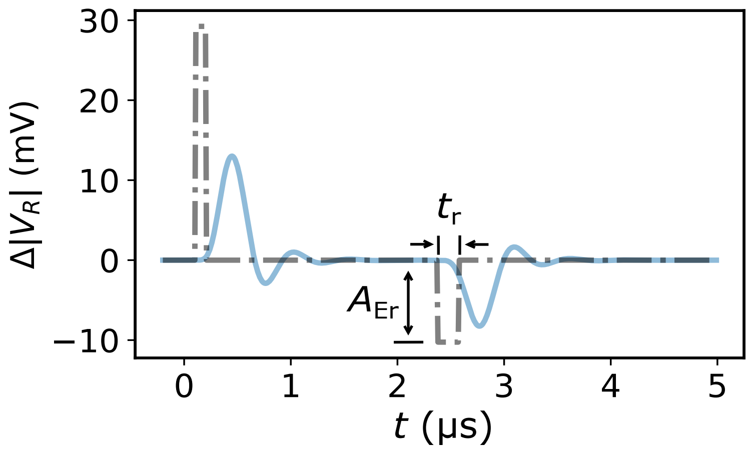

FIG. 8. **Simulation of infidelity for photoionisation detection.** The simulated $\Delta|V_{\mathrm{R}}|$-time trace with a reset time of 200 ns for applying (blue) and not applying (black dot dash line) the 2 MHz simulation low-pass filter.

transient jump has negligible impact on the fitting for the cycles in which the two events are well separated in time, such as the blue trace in Fig. 3a in the main text and the trace in Fig. 7(b).

In order to quantify the impact of laser-induce transient jump on the fitted ionisation time, t_{ion}, we evaluate the variation of t_{ion} as different values of A_{opt} are used in the fitting. First, we generate a noiseless trace with a set ionisation time, e.g., $t_{\mathrm{ion-set}} = 272$ ns, the two predetermined parameters, $t_{\mathrm{opt}} = 122$ ns and $A_{\mathrm{opt}} = 28.9$ mV, and a typical amplitude of the ionisation-induced falling edge, $A_{\mathrm{Er}} = -10.5$ mV. Second, the same noiseless trace is fitted multiple times with different values of A_{opt} ranging from 13.5 mV to 44.4 mV but with the correct values of t_{opt} and A_{Er}. These fittings give different results of t_{ion}. Based on the distribution of A_{opt} shown in Fig. 6(b), a distribution of t_{ion} is calculated and gives a standard deviation of $\delta_{\mathrm{fit}} = 28.1$ ns, as shown in Fig. 7(d). Finally, the above two steps are repeated for different set ionisation times, and the result, δ_{fit}, is plotted as a function of the set ionisation time in Fig. 7(e).

Altogether, each of these two parts contributes to an increase of the resolution of the fitted ionisation time. The final time resolution of the photoionisation detection is calculated as $2 \times \sqrt{\delta_{\mathrm{exp}}^2 + \delta_{\mathrm{fit}}^2}$ and is shown in Fig. 3b in the main text.

VII. INFIDELITY OF PHOTOIONISATION DETECTION

In the present study, ionisation events that last longer than 0.5 μs, the bandwidth limit, can be detected with near-unity fidelity, thanks to the high signal-to-noise ratio (SNR) of the RF signal. While the relatively short reset time of 70.9 ± 0.1 μs allows a fast detection repetition, this also leads to rapidly reset events, for example, 0.7 % of all ionisation events are expected to reset within 0.5 μs. Some of the rapidly reset events may result in falling-edge signals with an insignificant amplitude compared with the noise level, and are therefore not detected. In this section, we carry out simulations to evaluate the detection infidelity of the short-lived ionisation events.

First, a laser-induced rectangular pulse and an ionisation-reset pulse are generated with a reset time, t_{r}, as shown by the dash-dotted line in Fig. 8. Then, the 2 MHz low-pass filtering used in Fig. 6(a) is applied to produce a noiseless signal which is plotted as a blue line in Fig. 8. Next, we use a simple readout method of comparing the lowest value point in the trace to a threshold voltage. Because the falling and the rising edges of the rapidly reset ionisation events are both limited by the bandwidth, and consequently do not provide additional information for signal recognition. On the other hand, the contrast provided by the lowest value point is also sufficient for For long-lived ionisation events because of the high SNR of the ionisation-induced signal. Finally, we analyse the infidelity with this readout signal and the noise level of the measurement, and the results are shown in Fig. 4c in the main text.

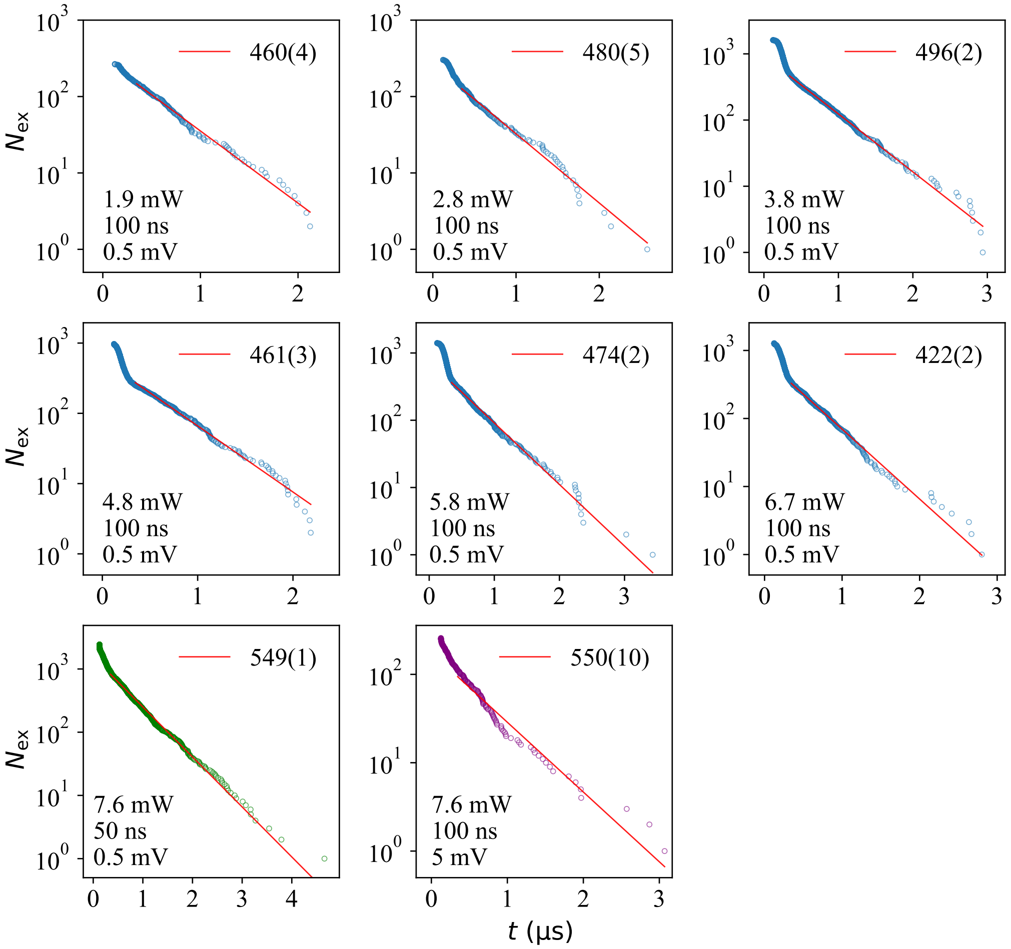

FIG. 9. **The excited state lifetime measurements.** The measurement conditions are specified at the bottom left region of each panel in order of laser power, pulse length and source-drain bias voltage from top to bottom. The number at the upper right region of each panel is the excited state lifetime of Er2 in nanoseconds from an exponential decay fitting.

VIII. THE EXCITED STATE LIFETIME OF Er2 UNDER DIFFERENT CONDITIONS

Figure 9 shows measurement results of the excited state lifetime of Er2 under the different experiment conditions. The fitting results are used to plot Fig. 3d in the main text.
